# The Promise and Peril of Mobile Phones for Youth in Rural Uganda: Multimethod Study of Implications for Health and HIV

**DOI:** 10.2196/17837

**Published:** 2021-02-02

**Authors:** Philip Kreniske, Alyssa Basmajian, Neema Nakyanjo, William Ddaaki, Dauda Isabirye, Charles Ssekyewa, Rosette Nakubulwa, Jennifer S Hirsch, Andrea Deisher, Fred Nalugoda, Larry W Chang, John S Santelli

**Affiliations:** 1 HIV Center for Clinical and Behavioral Studies New York State Psychiatric Institute and Columbia University New York, NY United States; 2 Heilbrunn Department of Population and Family Health Mailman School of Public Health Columbia University New York, NY United States; 3 Sociomedical Sciences Mailman School of Public Health Columbia University New York, NY United States; 4 Rakai Health Sciences Program Kalisizo Uganda; 5 Department of Epidemiology Johns Hopkins Bloomberg School of Public Health Baltimore, MD United States; 6 Division of Infectious Diseases Department of Medicine Johns Hopkins School of Medicine Baltimore, MD United States; 7 Pediatrics Vagelos College of Physicians and Surgeons Columbia University New York, NY United States

**Keywords:** adolescence, youth, mobile phones, cell phones, mobility, HIV, East Africa

## Abstract

**Background:**

In East Africa, where landlines are used by 1% of the population and access to the internet is limited, owning a cell phone is rapidly becoming essential for acquiring information and resources. Our analysis illuminates the perils and potential promise of mobile phones with implications for future interventions to promote the health of adolescents and young adults (AYAs) and to prevent HIV infection.

**Objective:**

The aim of this study is to describe the current state of AYAs’ phone use in the region and trace out the implications for mobile health interventions.

**Methods:**

We identified 2 trading centers that were representative of southern Uganda in terms of key demographics, proportion of cell phone ownership, and community HIV prevalence. We stratified the sample of potential informants by age group (15-19 years and 20-24 years), gender, and phone ownership and randomly sampled 31 key informant interview participants within these categories. In addition, we conducted 24 ethnographic participant observations among AYAs in the communities of study.

**Results:**

AYA frequently reported barriers to using their phones, such as difficulty accessing electricity. Nearly all AYAs used mobile phones to participate in the local economy and communicate with sexual partners. Phone use was frequently a point of contention between sexual partners, with many AYAs reporting that their sexual partners associated phone use with infidelity. Few AYAs reported using their phones for health-related purposes, with most getting health information in person from health workers. However, most AYAs reported an instance when they used their phone in an emergency, with childbirth-related emergencies being the most common. Finally, most AYAs reported that they would like to use their phones for health purposes and specifically stated that they would like to use their mobile phones to access current HIV prevention information.

**Conclusions:**

This study demonstrates how mobile phones are related to income-generating practices in the region and communication with sexual partners but not access to health and HIV information. Our analysis offers some explanation for our previous study, which suggested an association between mobile phone ownership, having multiple sexual partners, and HIV risk. Mobile phones have untapped potential to serve as tools for health promotion and HIV prevention.

## Introduction

### Background

In East Africa, where landlines are used by 1% of the population [1] and access to the internet is limited [2], owning a mobile phone is rapidly becoming essential for acquiring information and resources [1]. In the past 10 years, mobile phone ownership in East Africa doubled from 30% to over 70% [2], with recent analyses indicating similar trends in the Ugandan region of study [3]. Thus, mobile phones may present an opportunity for contacting and providing health information to adolescents and young adults (AYAs) and referring them to health care services [4-6]. However, ethnographic [7,8] and quantitative research in East Africa have also documented the association between mobile phones and sexual behaviors [3]. This has implications for health as East Africa is among the regions with the greatest number of new HIV infections in the world, with AYAs at greatest risk [9]. The objective of this study is to describe the state of AYAs’ phone use in a rural region of southern Uganda and identify the implications for HIV prevention and mobile health interventions.

### Perils of Mobile Phones

In Uganda, our recent cross-sectional analysis of the Rakai Community Cohort Study (RCCS) suggested that after adjusting for demographic characteristics, including socioeconomic status, people who owned mobile phones were more likely to have multiple partners than people who did not have mobile phones and that young women (15-24 years) who owned mobile phones were less likely to use condoms consistently, more likely to consume alcohol before sex, and more likely to be HIV positive [3]. However, the mechanism for this increased risk was not explained, and we called for further qualitative examination of the issue.

Previous ethnographic research has investigated the proliferation of mobile phones in relation to sexual intimacy and HIV risk. One ethnographic study found that university students in Tanzania used cell phones to maintain privacy in romantic relationships and to engage in transactional sex, defined as the exchange of sex for material resources or symbolic capital [10]. Ethnographic research with boarding school students in Kenya found that men gifted phones to female classmates expressly to schedule future meetings and engage in sexual relations outside of school [11]. These studies highlight the need to better understand the mechanisms connecting mobile phone ownership and sexual risk.

### Promise of Mobile Phones

The majority of mobile phone–based health interventions in low-to-middle-income countries (LMICs) leverage SMS text messaging [12,13]. A review found mobile phone–based interventions for adolescent sexual and reproductive health (SRH) in LMIC to be an effective way to reach young people, increase health knowledge, and achieve behavior change and noted a need for more research on security, confidentiality, and structural issues such as phone access [12]. More generally, several systematic reviews have shown that there is substantial evidence for the utility of mobile phone reminders to improve antiretroviral therapy adherence for people living with HIV in East Africa [14,15]. Studies in Uganda suggest that SMS text messaging is also a feasible and acceptable platform to support tuberculosis medication adherence [16,17] and testing [18,19]. Given the rapid rise in mobile phone ownership and the types of phones available and the importance of tailored interventions, there is a pressing need to examine the current state of mobile phones among AYAs in East Africa.

## Methods

### Data Sources

#### Background of the Research Setting

The greater Rakai region in southern Uganda is largely rural with dispersed trading centers. This paper focuses on the trading centers that have higher concentrations of AYAs and higher HIV prevalence than agricultural communities [20]. In both trading centers and agricultural communities, agricultural work is the main source of income and subsistence for most of the population [3]. The region borders Lake Victoria in the east and Tanzania in the south. The population is approximately 516,000, most people (57%) are aged between 0 and 17 years, with 19% aged between 18 and 30 years and 24.2% aged 31 years and older [21]. HIV prevalence from 2011 to 2013 ranged from 10% to 25%, depending on the community [20], and adjusted HIV incidence rate was 0.58 cases per 100 person years [9].

National data indicate that 73% of households have a mobile phone [22], which is comparable with the proportion of people in the rural region of the study who own phones [3]. However, only 16% of mobile phone users own smartphones [23]. In 2016-2017, the national unemployment rate was 9.7% and the median monthly income for employed residents in rural Uganda was UGX 150,000 (US $39.88) [24]. Ugandans who own mobile phones spend an average of UGX 14,500 (US $3.85) per month on voice calls [25], which is nearly 10% of the median monthly earnings for a rural resident. For smartphone users, 1 gigabyte of prepaid data generally costs UGX 10,265 (US $2.77) [23]. In addition, in 2018, the Ugandan parliament passed into legislation the over-the-top tax (OTT) of UGX 185 (US $0.05) per day to use social media services [26]. Although the cost of maintaining cell phone ownership in relation to median monthly earnings varies greatly based on region, rural residents generally endure a higher cost of cell phone ownership in relation to their income. Many mobile phone users use pay-as-you-go services [27], buying SIM cards, often from multiple networks [28], to pay for calls and SMS text messaging services when disposable income is available.

#### RCCS Quantitative Data

We used RCCS demographic data from 2018 to identify key informant interview (KII) participants. Beginning in 1994 and continuing to date, the RCCS records demographics and SRH responses from an open cohort of residents aged 15-49 years from 40 communities in the greater Masaka region of Uganda. The RCCS research design and procedures have been described in detail elsewhere [9,29].

In this study, the RCCS data set enabled our team to purposively sample participants for KIIs. On the basis of a series of meetings with local experts, field observations, and a review of RCCS data, we identified 2 trading centers that were representative of the region in terms of key demographics, including the proportion of AYAs to the total trading center population, distribution of occupations, proportion of cell phone ownership, and community HIV prevalence. We then stratified the sample of potential KII participants by age group (15-19 years and 20-24 years), gender, and phone ownership and randomly sampled KII participants within these demographic categories.

#### The Rakai Health Sciences Program’s Social and Behavioral Sciences Team

Social and Behavioral Sciences (SBS) team members began qualitative data collection in June 2018 and gathered the majority of data between January and May 2019. The SBS team members (authors 3-7) were fluent in English and Luganda (the most widely used language in the region). Most team members lived in the region of study and had 5 to 10 years of experience conducting qualitative research.

#### Semistructured KII

Drawing on narrative theory [30-32], the research team collaboratively designed and revised a series of KII questions that would elicit stories about participants’ lives. The narrative approach posits that people communicate their beliefs and practices through stories and that systematic analysis of stories can illuminate structural and social dynamics. For example, in this study, asking participants to tell a story about a time they could not use their phone might illuminate structural barriers, such as inconsistent network coverage, or social dynamics such as which members of society were permitted to own and use phones. Thus, in this study, the KII questions were designed to prompt AYAs to tell stories about their mobile phone practices as they related broadly to 4 main domains of interest: socializing practices, mobile phone use and barriers, behaviors with intimate partners, and health information–seeking behaviors. Multimedia Appendix 1 contains the full list of interview questions and probes.

#### Ethnographic Participant Observations

Ethnographic participant observation (EPO) involves engaging with and recording people’s daily lives [33]. Before conducting EPO, the SBS team created social maps of the communities of study with a focus on AYAs [34,35]. On the basis of these social maps and subsequent discussions, we identified 7 locations in which to conduct EPO: 1 hair salon, 1 boda (inexpensive and commonly used motorcycle taxi) stage (a place where boda boda drivers congregate and wait for customers), 2 bars, and 2 outdoor pool tables. At each location, the researchers identified an index case who served as the main contact and was present at each visit. SBS team members selected index cases who they determined to be regulars at the study site. SBS researchers visited each location 3 to 4 times over a 1-month period, spending approximately 2 hours per visit and observing and interacting with participants who they documented using field notes [36].

### Analysis

This study analyzes KII and EPO data using grounded theory to present detailed characterizations of how AYAs in peri-urban locations were using mobile phones with implications for health risks and future interventions [33,37]. The iterative process took the form of a data analysis spiral [38] such that following data collection, we organized the data, read and memoed emerging ideas, described and classified codes, developed and assessed interpretations, and finally created an account of our analysis in the form of this research report.

Authors 1 and 2 read all the KII and EPO field notes several times and compiled the data in the qualitative software program Dedoose [39]. Using Dedoose, author 1 wrote and discussed the initial memos with author 2 and then created preliminary codes. At this point, as there were increasing cases of the same codes, with few new codes emerging, author 1 and author 2 believed thematic saturation had been achieved [37,40]. Author 1 then discussed all preliminary codes with all authors in a series of meetings in New York and Uganda, and SBS team members (authors 3-7) provided additional memos and codes. Authors 1 and 2 refined the preliminary codes, and using 20% of the data, they achieved 90% interrater reliability.

### Ethical Considerations

Approvals for this study were obtained from the Research and Ethics Committee of the Uganda Virus Research Institute and the Uganda National Council for Science and Technology and from the Institutional Review Boards at Columbia University.

## Results

### Organizational Note on Results

In each section, we first present the general patterns identified in the KII and support these with key excerpts from the KII and, in some cases, EPO vignettes.

### Participants

We included both AYAs who owned at least one mobile phone (n=22) and AYA who did not own a mobile phone but did have access to a shared phone (n=8) in the KII (Table 1). Our intention in interviewing AYAs who did not own phones was to compare their beliefs and reported behaviors with those of AYAs who did own phones.

**Table 1:** Characteristics of 15- to 24-years-olds who participated in key informant interviews.

Characteristics		Values, n (%)	
		Men	Women
Number of interviews		14 (45)	17 (55)
**Mobile phone status**			
	Owns a mobile phone	10 (71)	13 (77)
	Does not own a mobile phone	4 (29)	4 (24)
**Age (years)**			
	15-19	7 (50)	6 (35)
	20-24	7 (50)	11 (65)
**Primary occupation**			
	Housework	0 (0)	4 (24)
	Unemployed	0 (0)	1 (6)
	Student	8 (57)	8 (47)
	Business	0 (0)	3 (18)
	Other (driver, carpenter, etc)	6 (43)	1 (6)

### Phone Use Practices

In our final KII sample, most of the participants owned at least one phone (n=17), with 6 participants owning 2 phones and 6 participants only having access to but not owning a phone, a common practice in the region [41]. In the cases where participants owned 2 phones, 1 was a *flip phone* or often referred to in the region as a *button phone* and 1 was a smartphone. A button phone can be used to make calls and send text messages and occasionally has a very basic camera, and according to AYAs, some button phone batteries last over a week on one charge. In contrast, smartphones are almost always equipped with cameras, can use apps, and are able to access the internet and specifically social media. However, smartphone battery life is limited and AYAs reported needing to charge their smartphones daily or sometimes twice a day. These different functionalities were important to AYAs, and therefore, a substantial proportion of AYAs owned 2 phones. Across age and gender, making calls was the most consistently reported phone practice, followed by money transfer and social media. In terms of differences by gender and age, men and older AYAs (20-24 years) more often reported using their phones for work, and young people (15-19 years) more frequently reported playing games.

AYAs from a range of professions viewed phone ownership as a necessity for conducting business. This included boda boda drivers, a motorcycle taxi driver, and hairstylists. One woman who was about 30 years old and a hairstylist described as follows:

I use my phone to get customers, they call on it, or someone can send me a picture of hair style and she says that she wants the same style. I use the phone to know more different styles for brides because it is my job to style brides...

For this hairstylist, the phone served as a critical tool for gathering information, such as *different styles for brides*, and for communicating with customers.

### Barriers to Using Phones

As illustrated in quote 1 in Table 2, the most frequent barrier to using a phone was the lack of electricity for charging. As noted in quote 2 in Table 2, other common barriers were lack of network coverage, lack of airtime, and the overall costs of owning and maintaining a phone.

**Table 2:** Barriers to using phones quotes.

Quote number	Topic	Quote
Quote 1	Electricity	“You know in Uganda electricity is not constant and you are not certain that electricity will be available tomorrow. So, you have to charge every day, yet we pay a lot of money for electricity.”
Quote 2	Network and cost	“The battery is weak and it gets low quite often. Another thing is network, sometimes the network is not good. I have a friend in Kaliro but usually when you are talking it loses network and I do not get what he is talking. Then also money for airtime, buying sms, it is expensive for me because you have to load SMS for a day if you want to communicate with a friend.”
Quote 3	Unwanted messages	“Unwanted text messages. Haha. They are my biggest problem. I get stressed out and it ruins my day when I get them. Then there are frauds who lie to us on the phone that we have won something when they want to take our money. It makes me want to throw away my phone. Also, stalkers who refuse to introduce themselves and leave you in suspense. Then the problems with phones is that even if you break up with someone, they can't leave you alone.”

Three participants noted that strangers calling them were an added burden to using their phones, as described in quote 3 in Table 2. More women than men described unwanted messages and calls from the opposite sex as a major problem related to mobile phones. In addition, 2 participants reported that the OTT tax [26] was a barrier to using their phone.

### Potential Perils of Mobile Phones

In response to the question *Do people ever get into trouble with their phones?,* the most common response was that sending explicit content could lead to trouble, as indicated in quote 4 in Table 3.

**Table 3:** Trouble with phones.

Quote number	Topic	Quote
Quote 4	Explicit content	“One man here in town shared his personal video clip with a sex worker on WhatsApp, and then she also shared it with others. This man was famous in town and he lost dignity because of this video clip.”
Quote 5	Trouble with sexual partners	“Conflicts happen in marriage because people cheat a lot using phones. They are always in touch with other extra marital partners...I saw it on WhatsApp on my friend’s phones. It was even within this town, some guy was touching himself on a video and sent it to the wife of his best friend and yet it’s the woman’s husband who sent a text to a phone number of some guy in his wife’s phone. The husband pretending to be his wife asked him to send a video of himself nude and he did. When he sent the video, the guy leaked it. The guy got embarrassed.”
Quote 6	Trouble with strangers	“A stranger may call you and ask you to meet them when they have other ‘programs’ and he may end up killing you. The other thing is when it comes to relationships, you can fall in love with someone you don’t know and they cause you harm, like taking you for ritual sacrifice or raping or killing you.”
Quote 7	Trouble with sexual partners and intimate partner violence	“I am a married woman. A phone can bring me problems if ‘kojja’ [uncle] that I work with admires me and asks me for my number. If I give it to him and he calls me when I am at home and he starts telling me that I look good among other things, my husband will become jealous, I will end up being beaten or we shall fight. He will start saying that I am promiscuous. The phone will have caused me problems.”

The next most frequent *peril* noted was potential trouble related to sexual partners. In fact, all of the women and half of the men we interviewed reported that they faced trouble related to mobile phones and their sexual relationships. One woman’s description of this trouble related to the themes of sexual partnership and sending explicit messages is included in quote 5 in Table 3.

*Trouble* related to mobile phones and sexual partners most often involved perceived infidelity, although in a few cases, this also included meeting new partners who were HIV positive and meeting new and physically dangerous partners, as described in quote 6 in Table 3. The ritual sacrifice noted in quote 6 may be more folktale rather than actual practice. However, intimate partner violence stemming from mobile phone communication was a concern for many women. For example, in quote 7 in Table 3, a 24-year-old woman described how calls to her mobile phone from other men stoked her husband’s jealousy and led to intimate partner violence.

#### Communicating With Sexual Partners

Nearly every participant reported using their phone to communicate and flirt with their sexual partners. Across age and gender, the most common way to communicate with partners was through phone calls, followed by texting, and less frequently social media. In quote 8 in Table 4, a 21-year-old man succinctly replied to the question *How would you make plans to meet your partner?* Likewise, in quote 9 in Table 4, a 20-year-old man explained how he made plans with his partner and how these plans depended on her mobile phone. An 18-year-old woman described a similar pattern, “He calls me and tells me where to meet him.”

A 20-year-old girl explained that despite the distance that separates her school from her partner’s home, they use their mobile phones to make plans and meet up, as detailed in quote 10 in Table 4. Describing a similar process of using mobile phones to overcome geographical and logistical obstacles, an 18-year-old man spoke about communicating with his partner and arranging a meeting with a partner who lives in a different community in quote 11 in Table 4.

The following passage demonstrates how our EPO at a boda boda stage corroborates these findings. A boda boda stage is a place where motorcycle taxi drivers gather and wait for customers, and extensive research has documented the hierarchy and organizational structure of boda boda stages in Uganda [42]. This boda boda stage, constructed using timber and roofed with tarpaulin, was overcrowded with motorcycles parked close to each other as if they were displayed for sale and was located at a junction where dirt roads branched off the main paved road. Author 5 spoke with a talkative young man, who was the vice-chairperson or the second in command below the chairperson of the boda boda stage. Dressed in tight black jeans with a black T-shirt and holding 2 phones, a smartphone and a basic phone, the vice-chairperson explained, “Here is my smartphone, I bought it for UGX 350,000 (US $100), I use it for WhatsApp and Facebook.” The vice-chairperson described how “most young people at boda boda stage use these smartphones purposely Okukwana Bakazi.” When asked to clarify, the vice-chairperson explained okukwana means getting a woman and starting a relationship with her. He continued, “There is no way you can connect with a woman without a phone.” Later, another boda boda driver supported this perspective and further explained that he used Facebook to “post messages using the smart phone. Sometimes these female sexual partners are in town.” This driver also showed his basic phone that he said he used to communicate with customers.

**Table 4:** Mobile phones for communicating with sexual partners.

Quote number	Topic	Quote
Quote 8	Call, text, and WhatsApp	“I would say in three ways. I would call her. I would text her or would go to WhatsApp and send her an audio on WhatsApp.”
Quote 9	Meeting my partner	“In most cases, I first call my sex partner to meet with her. I don’t see any other plan I would consider meeting my sex partner without a phone call. If her mobile phone number is switched off, I must wait until it is switched on.”
Quote 10	Back to school	“Now for example, we shall go back at school on 4th of February, for the first two weeks we are usually not busy because they are still registering and receiving students and we are waiting for results. So, over the weekend, I have the phone it is here (she shows it to the interviewer), I call him, or he calls me and tells me that I am going to come, and I also tell him that it is fine to come since we are mature.”
Quote 11	A partner in a different community	“I call her wherever she is or I can send her a message and tell her that I want you to come and see me next week. She can give me her program and tell me that I will not be able to make it. If she accepts, I send her transport and she comes then we meet. In case she comes, she spends time with me on that day and then she goes back. I give her transport then she goes back. We keep communicating over the phone.”

#### Mobile Phone–Specific Gift-Giving Practices

Nearly all the men in this study, but few women, reported giving gifts to their sexual partners. Given the breadth of the literature on transactional sex, we will focus our analysis on instances of mobile phone or mobile phone–related gift giving. Some men also reported giving their partner phones and airtime, but no women did so. After AYAs described gift-giving practices, we probed further and asked the gift givers if *they expected anything in return*. No participants indicated that they expected anything in return for these gifts.

Most participants did, however, report receiving gifts from their sexual partners. Many more women than men reported getting a phone or airtime as a gift from their sexual partner. It was also more common for older AYAs (20-24 years) than younger AYAs (15-19 years) to receive a phone. The practice of giving airtime was common for both older and younger AYAs. Author 5 noted how the vice-chairperson of the boda boda stage succinctly described one reason men buy phones for women, “It is true, the boda boda men buy phones for women to make it easier to communicate with them.”

When we asked *Does your partner expect anything in return*?, most respondents replied *no.* However, a few women and 1 man who received gifts felt that their partner expected something in return. In terms of age groups, no older AYAs responded *yes* to this question; however, a few younger AYAs responded *yes*. One 18-year-old married woman responded that “For him, he did everything thinking that we must have sex.” Another exchange with a young woman who was a student was as provided in Textbox 1.

**Exchange with a young woman who was a student.**

Author 7: *Okay do you think that your partner expects you to give him something because he has given you a gift?*

AYA: *Mmmm...yes...*

Author 7: *Okay...so what do you think he expects in return?*

AYA: *Mmm...hahaha...the “other thing.”*

Author 7: *Do you mean sex?*

AYA: *Yes that one hahaha.*

Author 7: *What makes you think he expects that?*

AYA: *I think it’s because for all that he does for, that is the way I can pay him back.*

A young single man who was a student replied as follows:

Okay, I would expect a bigger gift; this is one way to test her. However, for the case of our young women, I know that they will always expect something after offering small gifts.

Author 5, who conducted this interview, included in his field notes that the word *something* implied sexual relations. Both men and women considered gift giving to be a common practice with sexual partners. Interestingly, these responses suggest that some youths who received gifts believed that the gift giver expected sex in return for the gift. However, none of the AYAs who gave gifts reported that they expected sex in return for gifts they had given.

### Promise of Mobile Phones

To gather information about how phones may present a key opportunity for gaining health information, we asked participants the open-ended question, *How do you get health information?* Participants most frequently noted getting health information from the Rakai Project and other health workers. Television and radio were also frequently mentioned as sources of health information. In the region, bars and other small shops often display a television, which becomes a public gathering space. One 20-year-old student described her sources of health information as consisting of radio and television, as described in quote 12 in Table 5.

**Table 5:** Mobile phones and health information.

Quote number	Topic	Quote
Quote 12	Radio and television	“In most cases there is a program on Capital FM Radio, it is there at 8:00 and there is a doctor I always listen to. There are also some health tips on the radio and TV. Every day I have to listen to that doctor.”
Quote 13	Health worker call	“One health worker called me to come to the clinic and meet health workers. She told me to come and test for HIV. So, I left my work and went to the clinic.”
Quote 14	Not for health	“No, we do not usually think of using phones to get health-related services. Using phones to get health related information? Another girl who is a customer echoes, no we use our phone to call our men (laughing).”
Quote 15	Need for information	“The health information that I would like to access on my phone is to be able to know the ways in which I can protect myself from HIV.”
Quote 16	An emergency	“I fell sick, I did not have the energy to press the phone but during that time I was still staying with my wife. She brought the phone and I told her to check in my phone a health worker called, she called her and told her I am very sick, and I need quick medical attention. She asked her where I am at that time and my wife told her that I am at home, but I cannot walk. So, the health worker came from the health center to my home and gave me medication and the situation improved. However, if it was not for a phone, remember I could not walk and the wife was already in fear this could make me lose my life.”

Only 1 participant mentioned that school was a place where they received health information, and 1 participant noted that using her phone to search the internet was a source of health information. When we probed and specifically asked if people had *ever used their mobile phones to gather health information*, a little less than half of the participants said they had. A few participants described how they had used their phones to stay in touch with a health worker, and a couple of other participants described searching the internet for health information, as detailed in quote 13 in Table 5. However, it appeared rare for people to use their phones for health purposes, as a couple of young women in a hair salon explained in quote 14 in Table 5.

Although currently phones are rarely used to access health information, most participants reported that they would like to use their phones for health-related information in the future. Most frequently, participants wanted to use their phones to access information about HIV prevention, as one young woman succinctly explained in quote 15 in Table 5. Following HIV, participants also frequently noted that mobile phones could be used for gathering information about other diseases, family planning, and finding a doctor.

Finally, most participants spoke of a time when they needed to use their phone in an emergency. Of these, the most common emergencies were health related, and a number of women also specifically described using their phones in an emergency related to childbirth and labor, whereas a few participants also described other types of medical emergencies. One young man explained how a mobile phone was critical during his incapacitation from malaria, as described in quote 16 in Table 5. Many participants felt that their mobile phones were a valuable resource during health-related emergencies.

## Discussion

### Principal Findings

In a region where many roads are unpaved and people have limited economic and health resources, mobile phones are changing the ways that young people form social connections [5,43-45], and these changing social dynamics have implications for sexual relationships [3]. This study demonstrates how mobile phone ownership is critical for engaging in economic, social, and sexual behaviors in the Rakai region. In fact, nearly every participant used a mobile phone to flirt and communicate with sexual partners. This finding provides some insight in terms of a possible mechanism for the quantitative associations between owning a mobile phone and having a greater number of sexual partners and between phone ownership and positive HIV status for young women [3]. Finally, although few AYAs currently use their phones for health-related purposes, we show that there may be great potential for mobile phones to serve as tools for health promotion in the future.

### Potential Health-Related Perils

The psychological and social consequences of sending and receiving pornographic images and videos was one of the main perils of mobile phones cited by participants. In addition, for all of the women we interviewed, and many of the men, using a mobile phone caused trouble, such as issues of suspicion and jealousy, with their sexual partner. Women, in particular, reported that their male partners were suspicious of any phone use. For some women, the consequences of owning a phone outweighed the benefits, and these women chose to either get rid of their phones or conceal their phone ownership from their primary sexual partners.

It was also clear that mobile phones were frequently used to communicate with sexual partners. As one young man explained “there is no way you can connect with a woman without a phone.” In addition, consistent with previous research, AYAs frequently noted the need to move to pursue jobs or education [46-48]. Owning a mobile phone enabled these AYAs to maintain social and sexual relationships despite the geographical distance imposed by job searches and attending boarding school.

In line with previous ethnographic research, we found mobile phones were at times a currency in transactional sex. This was a complex dynamic and should be interpreted in the context of previous work that has cautioned against assuming that transactional sex is specific to African contexts and noted that relationships where there is an exchange of money may also have important affective and social dimensions [11]. We found that when AYAs gave mobile phone–related gifts, they never reported expecting anything in return. However, some AYAs who received gifts (eg, mobile phones or airtime) did *feel* there was an expectation that they engage in sexual behaviors after receiving a gift.

### Promise of Mobile Phones for Health Promotion

Consistent with previous literature, we found that phones were critical for engaging in social and economic interactions [1,5,45]. From hairstylists to boda boda drivers, a mobile phone was required to conduct daily business, and this was more common for older participants as compared with younger participants. In contrast, more younger than older AYAs reported using their phones to play games. This may be relevant for future interventions that could leverage interest in mobile phone–based games to disseminate health information for younger AYAs.

Of note, few AYAs reported ever using their mobile phones for purposes that might promote their health. However, most participants expressed interest in using their phones for health-related purposes in the future. This disconnect highlights a critical area for future researchers to address the health needs of AYAs in southern Uganda and perhaps in other resource-limited settings. AYAs reported an interest in using their phones to gather information about diseases and family planning. In fact, AYAs in the region currently have limited access to sexual health information [4]. Half of all pregnancies in Uganda are unintended, and the vast majority of these pregnancies (88%) occur among adolescents [4].

AYAs overwhelmingly stated that they would like to use their mobile phones to stay up to date on information about HIV prevention. HIV prevention is certainly a crucial and pressing issue in the region. As noted earlier, East Africa has the greatest number of new HIV infections in the world, with AYAs at the greatest risk [9]. In fact, AIDS is the leading cause of death for AYAs in the region and the second leading cause of death for AYAs worldwide [49,50]. In Uganda, people under 30 years comprise 75% of the total population [21], and despite extensive combination prevention efforts, the HIV incidence rate for AYAs remains high [9]. AYAs in the region are highly mobile [46-48], and evidence suggests that structural factors force AYAs to move and search for work after they leave school [46]. Mobility makes it difficult to track AYAs for recommended health care [51], and mobility is also associated with a greater risk of HIV infection [47,48]. Accordingly, our analyses suggest that mobile phones present an opportunity to reach these highly mobile AYAs [5,43,44,46].

Despite the potential promise for future mobile phone–based health interventions, critical barriers remain. Most notably, AYAs in the region had inconsistent access to electricity. To ameliorate this obstacle, some AYAs maintain 2 phones—1 button phone that can last up to 1 week after a single charge and 1 smartphone that can access apps and the internet but requires daily charging. Access to electricity is also a potential barrier for researchers seeking to design interventions that would require smartphones or tablets.

### Limitations

We believe that our KII coding achieved thematic saturation for the overall AYAs’ sample. However, given our relatively small sample (N=31), we were only able to offer limited insights regarding health risk and health promotion opportunities by age group (eg, 15-19 years vs 20-24 years). Thus, a limitation of this study is that we may not have achieved saturation among these subgroup categories [37,40]. It is also possible that within our strata (owning a phone and not owning a phone), further data collection could yield additional insights with implications for mobile phone–related health risks and intervention opportunities in the region. For example, future research could focus exclusively on AYAs who own phones to examine specific mobile phone health intervention components and approaches that would best meet their needs and address barriers such as intermittent internet and electricity access.

### Strengths

The use of purposive sampling from a population-based cohort allowed our research team to reliably and efficiently identify AYAs among the strata of focus (owning a phone and not owning a phone). Furthermore, by using multiple methods of data collection, KII and EPO, we were able to corroborate KII statements from the 3 strata through ecologically valid observations of behaviors in the community.

### Conclusions

AYAs’ developmental period is associated with the increased importance of peers, increased technology use, increased mobility, and initiation of sex [47,52,53]. A recent systematic review of digital innovations for HIV and sexually transmitted infections noted the need for research to tailor behavioral risk-reduction interventions to specific contexts and populations [13]. In southern Uganda, we found that technology use, mobility, and sexual behaviors were interrelated. Nearly all AYAs relied on mobile phones for making sexual partnerships, and in many instances, their phones allowed them to overcome significant geographic and logistical barriers to maintain long-distance sexual partnerships. Few AYAs used their phones to access health information, although most were interested in using their phones to access health information in the future, with specific enthusiasm for current information on HIV prevention. Given these factors, adolescence is a potentially critical developmental period for introducing preventive mobile phone–based interventions to provide AYAs in resource-limited settings with current and credible family planning and HIV prevention information and services.

## Appendix

Multimedia Appendix 1 Key informant interview cell phone qualitative interview guide.

## Abbreviations

ART: antiretroviral therapy
AYA: adolescents and young adult
EPO: ethnographic participant observation
KII: key informant interview
LMIC: low-to-middle-income countries
RCCS: Rakai Community Cohort Study
SBS: social and behavioral sciences
