# Supplementary material for: The Promise and Peril of Mobile Phones for Youth in Rural Uganda: Multimethod Study of Implications for Health and HIV
Source: J Med Internet Res. 2021 Feb 2;23(2):e17837. doi: 10.2196/17837 (PMC7886611; doi:10.2196/17837)
Supplement: Multimedia Appendix 1 [file jmir_v23i2e17837_app1.docx]

Appendix A

SSTAR Key Informant Interview Guide

**Notes to the interviewers regarding the purpose of the sets of questions included in italics*

*Question 1 is to check that the quantitative data is current in terms of occupation or schooling.*

1. What is your primary occupation (*Or* for students in what level of school are you currently enrolled?) (USE the demographic form)

-Q*uestions 2-8 will give us an idea of how the participant socializes and the ways they use or don’t use phones for socializing with friends and partners. We should remind them that all responses will be stored confidentially with only their study ID#:*

1. Where do you hang out with your friends? And what do you do for fun?
2. How do you make plans with friends? How do you know where to meet?
   - Probe: (If participant says they used a phone ask to see a recent call log or text, note content)
3. Do you have sexual partners? Where do you meet them? What are some things you do with them? (e.g. hookups at home, or hotels? Dates? where do they go, restaurants, movies, bars? Probe: do you ever drink alcohol before sex?
4. Do you own or have access to a phone?
5. What percent of your friends own a phone? (for those in school, probe: what percent of fellow students own phones?)
6. How many lines (Airtel, Warid, MTN, etc.) do you use? Do you use certain lines for certain purposes?
7. How do you make plans to meet with partners?
   - [If yes to own or access] probe: Do you use your phone to communicate with you partner?
   - (Probe: Can you describe those communications or show the researcher phone logs or text messages?)
8. Do your partners flirt with you, or how does your partner show that they like you?

Do you use your phone to flirt? If so, how many people are you flirting with on your phone this week? Probe: How many of these people have you met in person this month after flirting on the phone? Did you have vaginal sex after flirting over the phone? Probe: in the past year how many sexual partners have you had? Probe: In the past year have you always, sometimes, or never used condoms during sex?

- - [If yes to own or access] probe: Do any of your partners ever call you or send you messages to flirt? Pictures? **Mobile money?**
  - probe : can I see any?

1. How do you flirt with your partner or how do you show that you like them?
   - [If yes to own or access] probe: Do you ever call them or send them messages to flirt? Pictures?
   - Probe : can I see any?

*-We also want to understand how youth get phones, and the characteristics and uses of these phones:*

1. Does your partner ever give you gifts?
   - If yes, do you think your partner expects anything in return?
   - Probe: ever a phone or airtime minutes?
2. [If yes to own or access a phone ask Q9-16, if NO ask them to answer about **a friend- details bolded below**] Can you tell me the story of your phone (probe, how and when you got it, what you use it for – meeting people, socializing, how?)
   - [If no] Can you tell me the story of how you access a phone**? (or if no access can you tell me the story of your friend who has a phone?)**
   - When did you first get a phone or get access? **(for people with no phones or access: how did your friend get their phone?)**
   - Can you remember how you met people and socialized before you had a phone? How is it different now?

(**For people with no phones or access: ask: how do you meet people and socialize, has it changed since more people started getting phones?)**

1. Is your phone [or the phone you access or **friends phone**] a basic phone, or a [smartphone](https://www.pcmag.com/encyclopedia/term/51537/smartphone) (can your phone access Google, Facebook, Whatsapp)?
2. Do you have it with you? May I see it? (**skip if no phone)**

(note or ask model & service)

1. Which services can your (**or your friends**) phone (or the phone you access) perform? (eg. internet, money transfer – mobile or airtel money)
2. Which applications does your (**or** **friends)** phone have? (will need to specify during probing)
3. What are the most common things you (**or your friend)** do with your phone? (eg. calling, receiving calls, accessing radio, music, games, whatsapp, internet, etc)
4. What is the most important activity you (**or your** **friend)** use your phone for?
5. What were the last three things you (**or your friend**) did with your phone?
   - Can I see?
   - May I document?

*-Questions 18-23 will give us an idea of phones in the broader social context:*

1. How many of your friends have phones? None Few Many All
2. How did your friends get their phones?
3. How is phone ownership viewed by your peers? (Cool? Routine?)
4. What are the most common activities that your friends use their phones for?
5. What do parents think about their children owning or using phones?
6. Do people ever get in trouble with their phones? How? (sending pictures, flirting, late night romantic meetups?)

- Can you tell me of a scandal related to something that someone did with a phone, or something embarrassing?

*-Questions 24-28 give us an idea of the ways phones may be used for education:*

1. **If current student:** Do you use your phone for educational purposes in school?

**If not a student:** How do you think students might use their phones for educational purposes at school?

1. **If current student:** Tell me about how you use your phone for purposes not related to education while at school?
2. **If not a student:** How do you think students use their phones for purposes not related to education while at school?
3. Are phones allowed in school?
4. Should phones be allowed in school why, why not?

*-Questions 29-38 will give us an idea of how phones might be used for health purposes:*

1. Tell me about a time that you really needed to use a phone.
2. Tell me about one time you used your phone in an emergency, can you describe what happened? Probe: such as to contact the police, medical emergency services, or the fire station, or to alert people?
3. How do you keep in touch with your family?

- Probe for phone owners and access: Do you use your phone to communicate with your family. How do you do this?
  - (Probe: Can you describe those communications or show the researcher phone logs or text messages?)

1. How many of your household members have phones?

None Few Many All

Probe: Who has and who doesn’t? Who usually has airtime and who doesn’t?

Probe: Who let’s you borrow or use their phones or airtime?

1. How do you get health information? (locate clinics, contact doctor, check illness symptoms)
   - [If yes to own or access phones] probe: Do you use your phone to gather health information?

[0] Never [1] Yes, daily [2] Yes, weekly [3] Yes, occasionally

1. [If yes] Tell me a story about a time you used your phone to gather health information (e.g. medications, symptoms, clinic locations)?

- (Probe: Can I see the phone logs or text messages?)

1. Do you think mobile phones can be used to access health information? How?
   - Probe: Do you have suggestions for this process, what would you find useful? What type of health information would you like to be able to access? Or what do you lack?
2. [If yes to own or access phones Q30-32] Do you use your phone to gather other kinds of information (e.g. politics, news, weather)?

[0] Never [1] Yes, daily [2] Yes, weekly [3] Yes, occasionally

1. What other kinds of information do you get from your phone?
2. What challenges do you face with having/using a phone? (e.g. charging, theft, getting money for airtime/data, coverage/network, pressure from parents/school staff).
